# Supplementary figures and images for: Utilisation of the Prestwick Chemical Library to identify drugs that inhibit the growth of mycobacteria
Source: PLoS One. 2019 Mar 12;14(3):e0213713. doi: 10.1371/journal.pone.0213713 (PMC6414029; doi:10.1371/journal.pone.0213713)

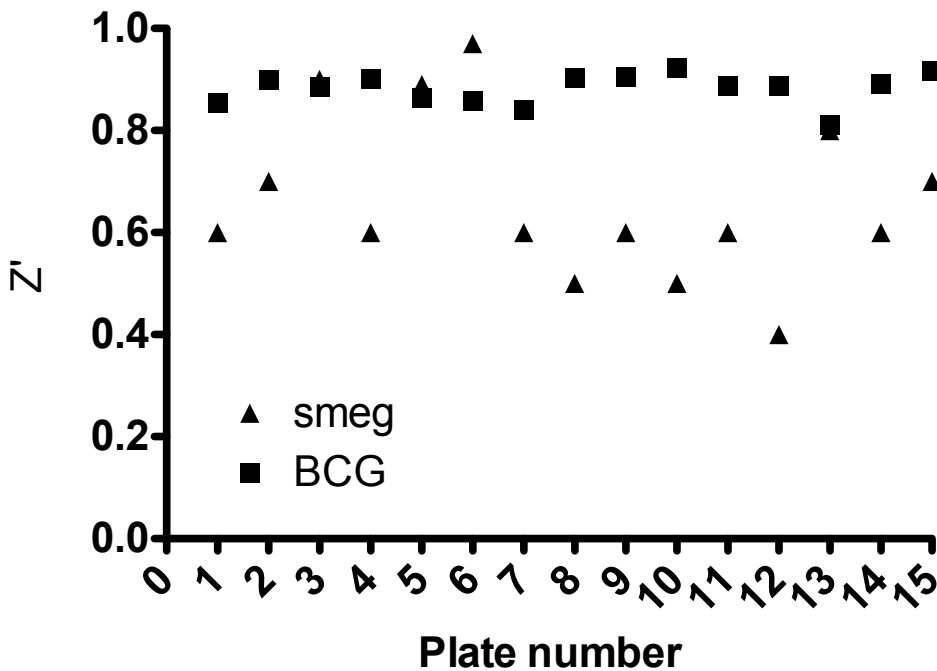

Supplement: S1 Fig — (PDF) [file pone.0213713.s001.pdf]

Increasing concentration of drug

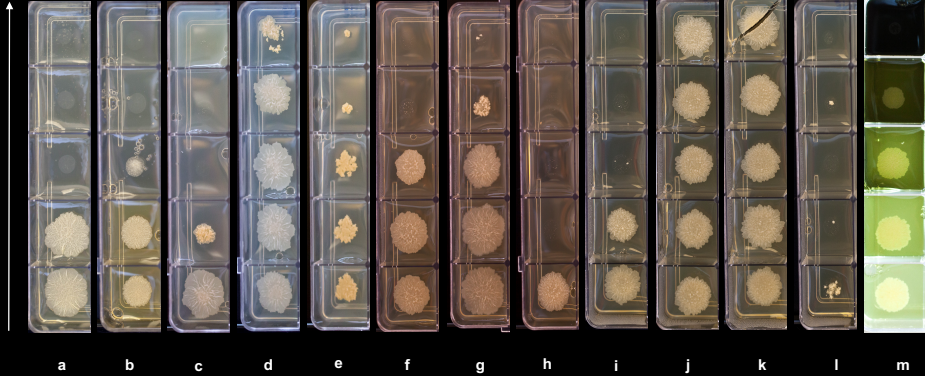

Negative control

Positive control

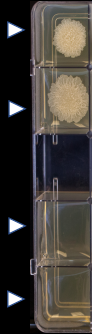

Supplement: S2 Fig — The solid MIC testing was performed against a. Sulocitidil, b. Auranofin, c. Raloxifen, d. Clomiphene citrate, e. Chlorhexidine, f. Fendiline hydrochloride, g. Tamoxifen citrate, h. Meclocyline sulfosalicylate, i. GBR12909, j. Nisoldipine, k. Sertraline, l. Toremifene, m. Apomorphine in the presence of a negative and positive control. (PDF) [file pone.0213713.s002.pdf]

Increasing concentration of drug

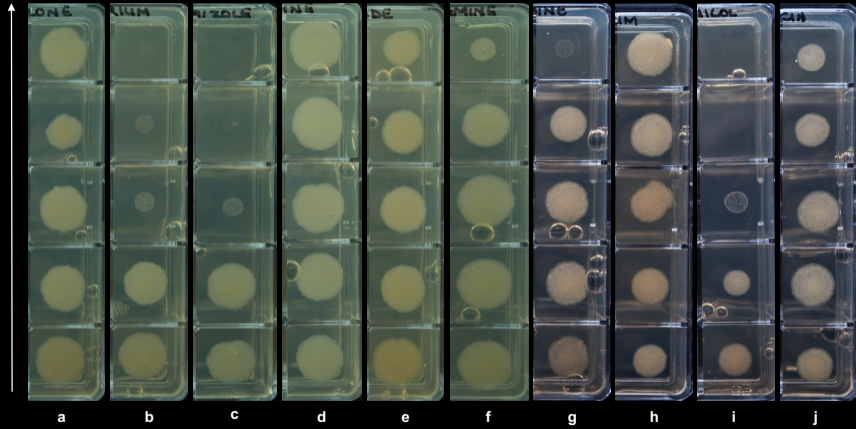

Positive control

Negative control

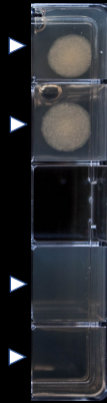

Supplement: S3 Fig — The solid MIC testing was done against a. Rosiglitazone, b. Pinaverium, c. Astemizole, d. Olopatadine, e. Glipizide, f. Tripelennamine, g. Pentamidine, h. Thonzonium, i. Florfenicol, j. Josamycin in the presence of a negative and positive control. (PDF) [file pone.0213713.s003.pdf]
