# Supplementary material for: Utilisation of the Prestwick Chemical Library to identify drugs that inhibit the growth of mycobacteria
Source: PLoS One. 2019 Mar 12;14(3):e0213713. doi: 10.1371/journal.pone.0213713 (PMC6414029; doi:10.1371/journal.pone.0213713)
Supplement: S4 Fig — The data shows a mean of three replicates. The OD values are derived from subtracting the OD from the test well which had been inoculated with M. tuberculosis from a blank well which had not been inoculated with bacteria. The error bars represent the standard error of the mean. (PDF) [file pone.0213713.s004.pdf]

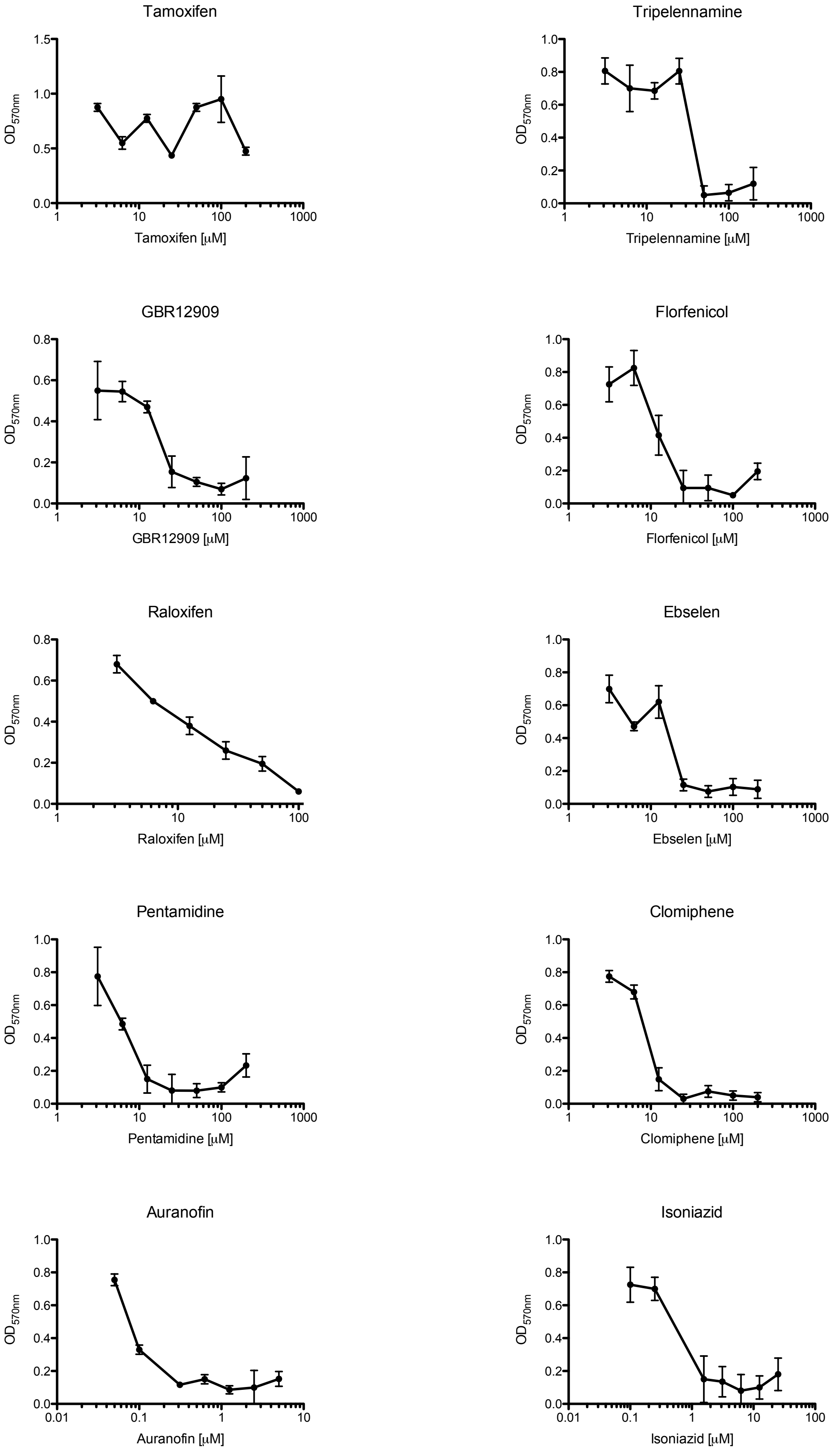
Tamoxifen
OD570nm
Tamoxifen [μM]
Tripelennamine
OD570nm
Tripelennamine [μM]
GBR12909
OD570nm
GBR12909 [μM]
Florfenicol
OD570nm
Florfenicol [μM]
Raloxifen
OD570nm
Raloxifen [μM]
Ebselen
OD570nm
Ebselen [μM]
Pentamidine
OD570nm
Pentamidine [μM]
Clomiphene
OD570nm
Clomiphene [μM]
Auranofin
OD570nm
Auranofin [μM]
Isoniazid
OD570nm
Isoniazid [μM]
